# Skull base invasive aspergillosis in a peritoneal dialysis patient: A rare and devastating complication

**DOI:** 10.1016/j.mmcr.2025.100758

**Published:** 2025-12-23

**Authors:** Nattapakorn Mai-on, Rubash Nath Yogi, Piyaporn Towangnang, Talerngsak Kanjanabuch

**Affiliations:** a Division of Nephrology, Department of Medicine, Faculty of Medicine, Chulalongkorn University, Bangkok, Thailand; b Department of Nephrology and Transplant Medicine, Shahid Dharmabhakta National Transplant Center, Nepal; c Peritoneal Dialysis Excellent Center, King Chulalongkorn Memorial Hospital, Bangkok, Thailand; d Center of Excellence in Kidney Metabolic Disorders, Faculty of Medicine, Chulalongkorn University, Bangkok, Thailand

**Keywords:** *Aspergillus flavus*, *Aspergillus mastoiditis*, Molecular identification, Peritoneal dialysis, Skull-base osteomyelitis, Voriconazole

## Abstract

*Aspergillus* mastoiditis with skull base osteomyelitis is an exceptionally rare but life-threatening infection in patients undergoing peritoneal dialysis (PD). We report a 60-year-old man with diabetic kidney failure on PD who developed invasive *Aspergillus flavus* mastoiditis progressing to skull base osteomyelitis. Despite early mastoidectomy, PD catheter removal, and voriconazole therapy achieving therapeutic levels, the infection advanced to involve the carotid sheath and parapharyngeal space, resulting in severe neurologic and systemic complications. Molecular sequencing confirmed *A. flavus*, excluding cryptic species such as *A. tamarii*. This case represents the first documented instance of skull base invasive aspergillosis in a PD patient and underscores the importance of early suspicion, species-level molecular confirmation, and multidisciplinary management in this formidable and rapidly progressive condition.

## 1 Introduction

Patients with kidney failure receiving peritoneal dialysis (PD) are particularly susceptible to infections, owing to uremia-associated immune dysfunction, frequent hospital administration, and a high burden of chronic comorbidities [1]. While bacterial peritonitis remains the most common infectious complication, fungal infections—especially extrapulmonary invasive aspergillosis—are exceedingly rare and carry high mortality. Among these, malignant otitis externa (MOE) with skull base osteomyelitis caused by *Aspergillus* species is an ultra-rare but devastating entity in the dialysis population. To date, only two cases have been reported in the literature—one from the UK involving concomitant *P. mirabilis* infection and another from Norway reported in Norwegian—both occurring in hemodialysis patients, with none previously documented in PD patients [2,3].

We describe a rare case of skull base invasive aspergillosis caused by *Aspergillus flavus* in a patient with diabetic kidney failure receiving PD. The infection progressed rapidly despite a combination of aggressive antifungal therapy and surgical intervention—underscoring the substantial diagnostic and therapeutic challenges encountered in this vulnerable population.

## 2 Case presentation

A 60-year-old Thai male monk with kidney failure secondary to diabetic kidney disease had been receiving nightly intermittent peritoneal dialysis (NIPD, 2 L × 5 exchanges) for two years. His comorbidities included poorly controlled type 2 diabetes mellitus, ischemic cardiomyopathy status post coronary artery bypass grafting with a left ventricular ejection fraction of 17 %, peripheral artery disease, prior ischemic stroke, post-parathyroidectomy secondary hyperparathyroidism, and severe protein–energy malnutrition.

He presented with a one-month history of left-sided otalgia and otorrhea without systemic symptoms. Two weeks later, he developed worsening otalgia, foul-smelling otorrhea, and new-onset left facial palsy. Temporal bone computed tomography (CT) revealed bony erosion involving the petrous portion of the temporal bone and adjacent skull base. Otoscopy demonstrated tympanic membrane perforation without granulation. Initial laboratory evaluation showed leukocyte count 9.64 × 10^3^/μL (Neu 72 %, Lym 11 %, Mono 6 %, Eos 9 %, Baso 1.3 %), hemoglobin 13.6 g/dL, hematocrit 44.1 %, and platelet 145 × 10^3^/μL. Chemistry results included Cr 6.1 mg/dL, Na 137 mmol/L, K 4.4 mmol/L, Cl 103 mmol/L, CO_2_ 25 mmol/L, AST 31 U/L, ALT 44 U/L, ALP 136 U/L, Ca 8.5 mg/dL, P 3.5 mg/dL, and Alb 2.3 g/dL.

A left mastoidectomy with facial nerve decompression was performed on July 27, 2024 (day 0). Surgical tissue was submitted for diagnostic evaluation. Portions were processed for histopathology, while parallel samples underwent Gram staining, acid-fast bacilli staining, modified acid-fast bacilli staining, and potassium hydroxide preparation, all of which were negative. Tissue specimens were cultured on blood agar, chocolate agar, and MacConkey agar for bacterial pathogens; on Sabouraud dextrose agar (SDA) and potato dextrose agar for fungal organisms; and on Loewenstein–Jensen medium and mycobacterial growth indicator tube culture system for mycobacteria. Polymerase chain reaction testing for *Mycobacterium tuberculosis* was also performed and yielded negative results.

**Fig. 1:**
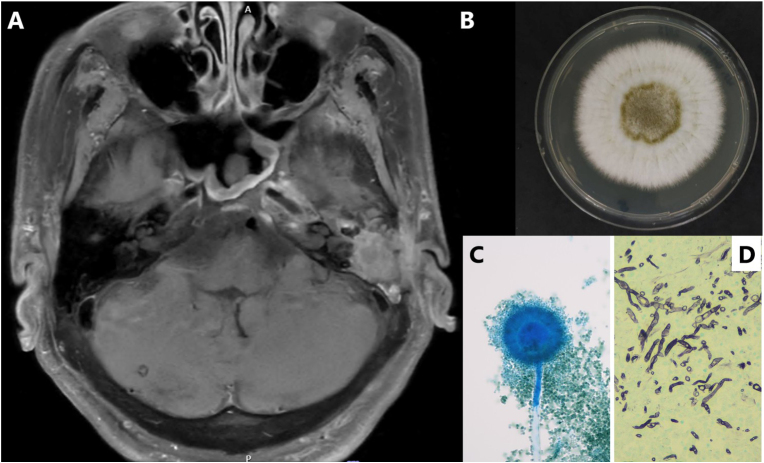
**(A)** MRI of the skull base demonstrating progressive osteomyelitis of the left petrous temporal bone with extension into the nasopharynx, carotid sheath, parapharyngeal space, and prevertebral muscle. **(B)***Aspergillus flavus* colony on Sabouraud dextrose agar after 7 days of incubation, exhibiting a velvety yellow-green surface with pale reverse pigmentation. **(C)** Lactophenol cotton blue mount (400 × ) showing rough conidiophores, biseriate phialides, and globose conidia characteristic of *A. flavus*. **(D)** Histopathology of mastoid tissue demonstrating tissue-invasive septate hyphae on Gomori methenamine silver (GMS) stain (200 x).

Empirical intravenous piperacillin–tazobactam was started, followed by oral ciprofloxacin after discharge. Bacterial cultures remained negative; however, fungal culture grew *A. flavus*. After 7 days of incubation on SDA, colonies exhibited a velvety, yellow-green surface with pale reverse pigmentation (Fig. 1B), and lactophenol cotton blue mounts demonstrated rough conidiophores, biseriate phialides, and globose conidia (Fig. 1C). Upon receipt of the pathology report confirming invasive fungal disease (Fig. 1D), the patient was readmitted for definitive management.

Molecular sequencing performed on the cultured isolate confirmed *A. flavus* using four primer sets—SSU (18SF–18SR), ITS (ITS1–ITS4), and LSU (NL1–NL4; LR0R–LR7) [[4], [5], [6], [7]]—each demonstrating 100 % query coverage and ≥99.7 % sequence identity (Table 1). DNA was extracted using the magLEAD® 12gC system (Precision System Science, Japan) according to the manufacturer's protocol with minor modifications. The sequences were deposited in GenBank (OL655454.1, KJ999737.1, ON231619.1, HQ395773.1). Antifungal susceptibility testing, performed according to the CLSI M38-A3 broth microdilution method [8], showed MIC values of 1 μg/mL for amphotericin B, 0.5 μg/mL for voriconazole, >16 μg/mL for itraconazole, and 0.5 μg/mL for posaconazole and isavuconazole, interpreted using EUCAST ECOFFs for *A. flavus* [9].

**Table 1:** Molecular identification of *Aspergillus flavus* from mastoid tissue culture.

Organism	Primer Set	Score	Query Cover	Identity	Accession No.
*A. flavus*	SSU (18SF–18SR)	778/778	100 %	100 %	OL655454.1
*A. flavus*	ITS (ITS1–ITS4)	909/909	100 %	100 %	KJ999737.1
*A. flavus*	LSU (NL1–NL4)	946/946	100 %	100 %	ON231619.1
*A. flavus*	LSU (LR0R–LR7)	1982/1982	100 %	99.72 %	HQ395773.1

Voriconazole therapy was initiated with a loading dose of 6 mg/kg twice daily on day 7, followed by 4 mg/kg twice daily administered orally (body weight 38 kg; total daily dose 200–400 mg), with dosing adjustments guided by therapeutic drug monitoring. The trough level on day 5 was 3.2 μg/mL. Despite adequate drug exposure, serial CT and magnetic resonance imaging (MRI) showed progressive skull base osteomyelitis with extension into the nasopharynx, carotid sheath, parapharyngeal space, and prevertebral muscle (Fig. 1A). Neurosurgical intervention was deemed unfeasible.

The patient subsequently developed focal seizures due to local invasion, followed by septic and cardiogenic shock requiring intensive care unit admission, vasopressor support, and continuous kidney replacement therapy. His course was further complicated by *Clostridioides difficile* colitis and transaminitis, necessitating temporary interruption of antifungal therapy.

Given relentless disease progression, lack of surgical options, and the patient's deteriorating condition, a multidisciplinary team—nephrology, infectious diseases, otolaryngology, and neurosurgery—reached a consensus to transition care toward comfort measures and end-of-life support.

## 3 Discussion

This case highlights a rare and devastating manifestation of extrapulmonary invasive aspergillosis: mastoiditis with skull base osteomyelitis caused by *A. flavus* in a patient receiving PD. While *Pseudomonas aeruginosa* remains the leading pathogen in MOE, this infection extended beyond the external auditory canal to involve the mastoid and skull base—features more consistent with invasive fungal mastoiditis rather than MOE alone [10,11].

Patients on PD, particularly those with diabetes mellitus, malnutrition, and repeated broad-spectrum antibiotic exposure, are predisposed to opportunistic fungal infections due to uremia-associated immune dysfunction [1,12]. Although fungal peritonitis is the most recognized invasive fungal complication in PD, non-peritoneal invasive mold infections remain rare but clinically significant. The MycoPDICS registry further demonstrated that filamentous mold infections in PD patients, while uncommon, are associated with high mortality and a high likelihood of transition to hemodialysis [13].

Accurate species identification is vital. *Aspergillus tamarii*, a cryptic species, has previously been misidentified as *A. flavus* by morphology alone in PD-related peritonitis [14]. In our case, molecular sequencing of ribosomal DNA using four validated primer sets confirmed *A. flavus* with high confidence, preventing potential misclassification and ensuring appropriate antifungal selection.

Clinically, *Aspergillus* mastoiditis often mimics chronic bacterial otitis, presenting with persistent otalgia, otorrhea, and, in advanced stages, cranial nerve involvement such as facial palsy. Imaging that demonstrates bony erosion of the petrous temporal bone or skull base should prompt suspicion for fungal osteomyelitis. In this case, diagnosis was established through histopathology demonstrating septate hyphae, compatible culture growth, and definitive species-level molecular identification.

Management of invasive *Aspergillus* mastoiditis requires timely surgical debridement and systemic antifungal therapy supported by susceptibility testing and therapeutic drug monitoring. Voriconazole is considered first-line therapy due to its potent activity, superior bone and central nervous system (CNS) penetration, and demonstrated clinical advantage over amphotericin B [[15], [16], [17]]. Although voriconazole therapy in this patient achieved therapeutic plasma levels, the disease progressed aggressively, reflecting the challenges of treating advanced osteoinvasive disease where pharmacologic therapy alone may be insufficient.

Once fungal osteomyelitis extends to the skull base, carotid sheath, and parapharyngeal or prevertebral compartments, surgical intervention becomes technically unfeasible. In such circumstances, multidisciplinary collaboration—encompassing otolaryngology, infectious diseases, nephrology, neurosurgery, and palliative care—is essential for individualized decision-making and, when appropriate, transition to comfort-focused care.

This case underscores several key principles: (1) Fungal mastoiditis should be considered in PD patients with refractory otitis, particularly when cranial neuropathies or bony erosion are present; (2) Species-level identification using molecular diagnostics is crucial to avoid misclassification and guide targeted therapy; (3) Voriconazole remains the preferred antifungal agent, but optimal outcomes depend equally on early diagnosis and feasible surgical source control; and (4) Multidisciplinary management is indispensable in advanced disease.

Ultimately, invasive A*spergillus* mastoiditis in PD patients, although rare, can be rapidly progressive and life-threatening. A high index of suspicion, early diagnostic evaluation, and coordinated care are essential to improve outcomes in this vulnerable population.

## CRediT authorship contribution statement

**Nattapakorn Mai-on:** Writing – review & editing, Writing – original draft, Data curation, Conceptualization. **Rubash Nath Yogi:** Writing – review & editing, Data curation. **Piyaporn Towangnang:** Writing – review & editing, Data curation. **Talerngsak Kanjanabuch:** Writing – review & editing, Validation, Supervision, Conceptualization.

## Conflict of interest

This research was generously supported by the Health Systems Research Institute (HSRI), Thailand [Grant No. 68-109], and the Thailand Science Research and Innovation Fund, Chulalongkorn University, Thailand [Grant No. HEA_FF_68_018_3000_002]. TK has received consultancy fees from VISTERRA and AstraZeneca as a country investigator, a research funding from the National Research Council of Thailand. He has also received speaking honoraria from AstraZeneca, Alexion Pharmaceuticals Inc., Fresenius Medical Care, and Baxter Healthcare. The remaining author declared no commercial or financial relationships that could be construed as potential conflicts of interest.
